# A LOOK INSIDE THE MISSOURI NURSING HOME COVID-19 EXPERIENCE

**DOI:** 10.1093/geroni/igac059.744

**Published:** 2022-12-20

**Authors:** Amy Vogelsmeier, Lori Popejoy

**Affiliations:** University of Missouri, Columbia, Missouri, United States; University of Missouri, Columbia, Missouri, United States

## Abstract

The COVID-19 pandemic exposed the vulnerabilities of US nursing homes to manage widespread viral outbreaks including an ill prepared/under-resourced workforce, a physical environment not conducive to infection prevention or management, and isolation from community emergency response planning. In this session, we will share real-life, real-time experiences of diverse Missouri nursing homes as they responded to the COVID-19 pandemic. We will also report on emerging data about the impact of nursing homes’ pandemic response on resident outcomes. Strategies such as community-based efforts to respond to resource scarcity, and creative workforce solutions to address staffing needs, will be shared. Critical next steps should focus on the implementation of community coalitions to create sustainable healthcare partnerships at the local and state level and enhanced workforce solutions that include registered nurses and advanced practice registered nurses working within nursing homes to guide clinical care and infection prevention and management strategies.

